# Analytical Methods for Oxalate Quantification: The Ubiquitous Organic Anion

**DOI:** 10.3390/molecules28073206

**Published:** 2023-04-04

**Authors:** Bryan Misiewicz, Donald Mencer, William Terzaghi, Adam L. VanWert

**Affiliations:** 1 Department of Pharmaceutical Sciences, Nesbitt School of Pharmacy, Wilkes University, Wilkes-Barre, PA 18766, USA; 2 Department of Chemistry and Biochemistry, Wilkes University, Wilkes-Barre, PA 18766, USA; 3 Department of Biology and Earth Sciences, Wilkes University, Wilkes-Barre, PA 18766, USA

**Keywords:** oxalate, oxalic acid

## Abstract

Oxalate is a divalent organic anion that affects many biological and commercial processes. It is derived from plant sources, such as spinach, rhubarb, tea, cacao, nuts, and beans, and therefore is commonly found in raw or processed food products. Oxalate can also be made endogenously by humans and other mammals as a byproduct of hepatic enzymatic reactions. It is theorized that plants use oxalate to store calcium and protect against herbivory. Clinically, oxalate is best known to be a major component of kidney stones, which commonly contain calcium oxalate crystals. Oxalate can induce an inflammatory response that decreases the immune system’s ability to remove renal crystals. When formulated with platinum as oxaliplatin (an anticancer drug), oxalate has been proposed to cause neurotoxicity and nerve pain. There are many sectors of industry that are hampered by oxalate, and others that depend on it. For example, calcium oxalate is troublesome in the pulp industry and the alumina industry as it deposits on machinery. On the other hand, oxalate is a common active component of rust removal and cleaning products. Due to its ubiquity, there is interest in developing efficient methods to quantify oxalate. Over the past four decades, many diverse methods have been reported. These approaches include electrochemical detection, liquid chromatography or gas chromatography coupled with mass spectrometry, enzymatic degradation of oxalate with oxalate oxidase and detection of hydrogen peroxide produced, and indicator displacement-based methods employing fluorescent or UV light-absorbing compounds. Enhancements in sensitivity have been reported for both electrochemical and mass-spectrometry-based methods as recently as this year. Indicator-based methods have realized a surge in interest that continues to date. The diversity of these approaches, in terms of instrumentation, sample preparation, and sensitivity, has made it clear that no single method will work best for every purpose. This review describes the strengths and limitations of each method, and may serve as a reference for investigators to decide which approach is most suitable for their work.

## 1. Introduction

**Figure 1:**
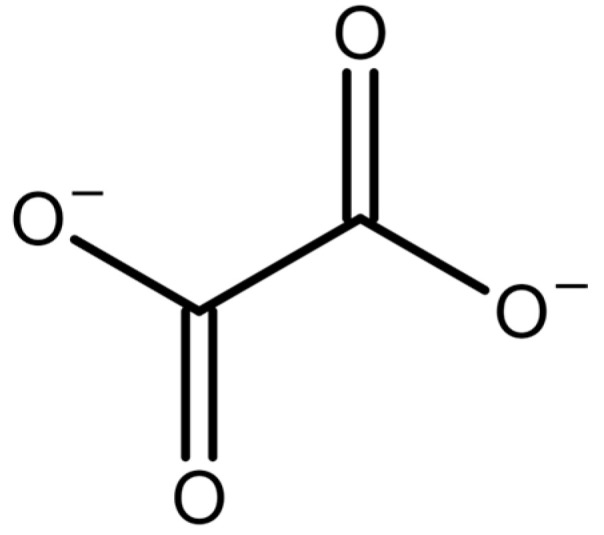
Structure of the oxalate ion.

Oxalate is a divalent organic anion (Figure 1) that plays a role in a wide variety of fields. It can be derived from plant sources, formed as a byproduct in some physiological reactions, and is even formulated with some drugs currently on the international market. It is best known as a major component of kidney stones (nephrolithiasis), but also plays a role in oxidative stress and inflammation in humans. It is also an important compound in plant physiology and defense [1]. Due to its diverse roles and impact on society, there has been great interest in quantifying oxalate for many years. Below, we provide more detail on various fields where oxalate is involved, and then describe the analytical methods used for quantifying oxalate.

### 1.1. Oxalate in Clinical Science

The incidence of nephrolithiasis in the United States is on the rise and is currently estimated to be similar to the prevalence of diabetes. The incidence of nephrolithiasis is 10.6% in men and 7.1% in women [2], whereas the prevalence of diabetes is 10.5% [3]. Calcium oxalate is the cause of over 70–80% of patients presenting with renal stones [2,4]. Oxalate is found in human blood at levels typically around 1–5 µM; however, the oxalate level in urine is typically 100 times higher. The supersaturation of renal ultrafiltrate and urine with oxalate is crucial for the formation of renal stones, which usually attach to subepithelial plaques (Randall’s plaques) or apatite deposits in the collecting ducts. Oxalate is passively absorbed in the human gut and also absorbed via carrier-mediated mechanisms [5]. Besides the diet, oxalate is formed in humans by the liver as an end product in glyoxylate metabolism by hepatic peroxisomes. Dysfunction of the glyoxylate metabolic pathway is the cause of primary hyperoxaluria type 1, which is a rare autosomal recessive disorder [6,7]. Dysfunction of SLC26A6 (also known as PAT1), a secretory intestinal transporter, has been correlated with increased urinary excretion in mice. SLC26A6 dysfunction has also been shown to cause a net increase in oxalate absorption [8]. Increased blood oxalate causes an increased renal burden and urinary excretion. Studies show that patients who have undergone malabsorptive bariatric surgery, especially Roux-en-Y gastric bypass, approximately double their risk of nephrolithiasis [9]. A link between oxalate and the renin–angiotensin system pathway has also been found showing that angiotensin II can increase intestinal secretion of oxalate, although renal insufficiency may be necessary to induce this interaction [5,10].

### 1.2. Inflammation/Immunology

Exposure of renal epithelial cells to oxalate can incite an inflammatory response via an increase in reactive oxygen species as well as a decrease in glutathione levels. Oxalate exposure has also been shown to impair DNA synthesis and cause cell death. Oxalate impairs monocytes’ ability to differentiate into macrophages, which are important for renal crystal removal. Increased dietary intake of oxalate has been shown to alter immune responses in healthy patients [2].

### 1.3. Drugs and Nutrients Containing Oxalate or Metabolized to Oxalate (Oxaliplatin, Vitamin C)

**Figure 2:**
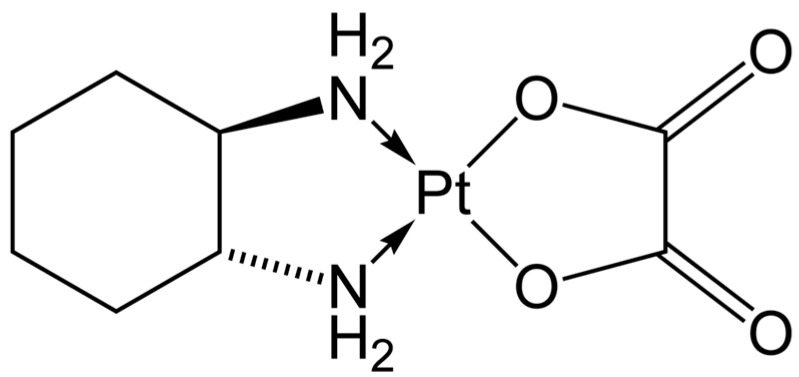
Structure of oxaliplatin.

Oxaliplatin is a platinum-containing anticancer medication (Figure 2) commonly used to treat a variety of cancers including breast, colorectal, and lung cancer. Once oxaliplatin is metabolized by the body, oxalate ions are one of two major metabolites that are formed. These oxalate ions are linked to negative side effects of the drug including severe nerve pain characterized by allodynia (hypersensitivity to painful stimuli) [11]. Oxalate is hypothesized to cause this neurotoxicity through two mechanisms: chelation of calcium by oxalate, which affects calcium-sensitive voltage-gated sodium channels; and an indirect action on voltage-gated sodium channels where oxalate alters intracellular calcium-dependent regulatory mechanisms [12]. Furthermore, oxalate has also been proven to decrease the maximum sodium current peak similarly to the decrease seen from oxaliplatin.

Ascorbic acid (vitamin C) is an essential component of the human diet as it cannot be synthesized endogenously. It plays important roles in several physiological functions including the synthesis of neurotransmitters and collagen, protein metabolism, immune function, and nonheme iron absorption, and it is also an antioxidant [13]. The breakdown of ascorbic acid by the body results in oxalate formation. Although additional studies are warranted, there have been multiple reports of ascorbic acid increasing a person’s risk of kidney stones [14]. One study from Sweden showed that supplemental ascorbic acid (around 1000 mg) intake doubled the risk of kidney stones [15].

### 1.4. Oxalate in Food Science

Calcium oxalate crystals are found in all taxonomic levels of photosynthetic organisms. Plants such as spinach, rhubarb, and tea have high levels of oxalate [6]. Chocolate derived from tropical cacao trees, strawberries, nuts, wheat bran, sweet potatoes, black olives, and most dry beans are also high in oxalate [5,16]. There have been problems reporting the oxalate content of foods due to inaccuracies of the analytical methods used. Of note, variability in oxalate between food samples also contributes to the challenge of accurately measuring oxalate. For example, changes in growth conditions, plant variety, and developmental stage of the plant are known to affect oxalate content [16]. Due to this variability, achieving a broad linear range for quantifying oxalate is essential.

### 1.5. Relevance of Oxalate in Plants/Fungi

Although the role of oxalate in plants has not been completely elucidated, it has been suggested that oxalate is involved in seed germination, calcium storage, detoxification, structural strength, and repelling insects [16]. Calcium oxalate is also important in plants to protect against herbivory and phytotoxins [17,18]. Many fungal pathogens secrete oxalate when they attack plants in order to weaken the plant cell walls to facilitate their degradation by fungal enzymes [19].

### 1.6. Oxalate in Industrial Processes

Oxalate is found in, or produced from, some household and industrial products/processes. For example, ethylene glycol is a chemical commonly used in antifreeze and coolant. There have been multiple case reports confirming that ethylene glycol toxicity causes hyperoxalemia, hyperoxaluria, and calcium oxalate crystal formation in the urine because oxalate is a metabolic product of ethylene glycol [20]. SerVaas Laboratories Inc. reports that the core ingredient in Bar Keepers Friend, a common cleaning product, is oxalic acid [21]. In alumina refineries, where bauxite ore is refined to alumina (Al_2_O_3_), sodium oxalate is a major impurity that coprecipitates with aluminum hydroxide. This sodium oxalate is then removed to both remove the impurity from the constantly recycled processing liquor and to recover sodium [22]. Calcium oxalate is troublesome in the pulp (paper) industry. Oxalic acid is found naturally in wood and, as it goes through the bleaching process, it deposits on machinery, decreasing product quality and profitability [23].

### 1.7. Need for a Review on the Current State of Analytical Methods for Oxalate/Oxalic Acid

An updated review of analytical methods for quantifying oxalate/oxalic acid is timely, as new approaches have been reported as recently as this year. Enhancements in sensitivity have been realized, taking advantage of modern instrumentation. Moreover, new colorimetric or fluorimetric methods have been developed. We review the strengths and limitations of both modern and older methods. The table provided in this review gives a comprehensive account of figures of merit (i.e., limit of detection, limit of quantification, linear range, recovery, and precision), approaches to sample preparation, modes of detection of oxalate/oxalic acid, and noteworthy strengths and limitations.

## 2. Methods for Detection and Quantification of Oxalate

Many diverse methods for detecting and quantifying oxalate have been reported over the past four decades. De Castro noted that a variety of methods were developed leading up to 1988, many of which have been improved and are used, in some form, currently [24]. The updated methods are first introduced in this review, and then more detailed descriptions with references follow. A tabular summary, including limits of detection and quantification, and other figures of merit, is provided in Table 1.

Enzyme-based methods employing oxalate oxidase have realized a niche in commercial oxalate detection kits due to their simplicity and relatively common instrumentation for reading samples. There appears to be some promise in conjugating oxalate-degrading enzymes with solid supports, as they can be washed and reused. From a commercialization perspective, it may be useful to develop a device with oxalate oxidase coupled to beads or a monolithic solid in order to circumvent some sample preparation steps associated with enzyme dilution. Alternatively, success has been achieved using sensors based on the injection of the recognition element (SIRE) approach, wherein small quantities of oxalate oxidase are added for one-time use.

Liquid chromatography–mass spectrometry (LC-MS) has historically not been a method of choice for oxalate due to its low sensitivity compared to other methods, and compared to other analytes using LC-MS. Perhaps the primary advantage is the more confident identification of oxalate using MS/MS, which may be acceptable if the oxalate content is above the limit of quantification. More recent reports have demonstrated improved sensitivity with LC-MS/MS, giving this approach a potential role in laboratories with access to the instrumentation. GC-MS has also not realized much utility in oxalate detection, possibly due to the time required for derivatization. However, investigators continue to explore GC-MS methods for oxalate detection.

The most sensitive oxalate detection methods to date are conductivity-based. These approaches do, however, often require corrections based on bulk solution conductivity changes, and loss of linearity at higher oxalate concentrations. In addition, the separation columns/capillaries coupled with electrochemical detection are often susceptible to fouling when real food or other biological samples are tested.

Finally, indicator displacement methods wherein oxalate turns on or turns off fluorescence, or alters UV absorbance, can be rapid and instrumentally simple, but are limited by the lack of specificity of the indicator.

Therefore, to determine which methodology is most suitable for quantifying oxalate, one must answer several questions first: What sensitivity is needed? What linear range is needed? How complicated is the sample matrix and what is the likelihood of interfering compounds? What instrumentation is available? What expertise is needed to employ the method? It is clear that no single method will be suitable for every investigation. A summary of the different approaches is presented in the following sections.

### 2.1. Enzyme-Based Methods

Enzyme-based detection of oxalate has been employed for several decades, and is used in several commercially available kits. In 1994, oxalate oxidase was immobilized in a column, where it was used to metabolize urine-borne oxalate into hydrogen peroxide and carbon dioxide [25]. In this flow-injection analysis method, chemiluminescence was measured from the reaction of hydrogen peroxide with luminol and hexacyanoferrate (III). The limit of detection was 34 µM (2.99 µg/mL), and linearity was confirmed up to 500 µM. The advantage of this method is that oxalate oxidase is specific for oxalate as a substrate; however, the disadvantage is that L-ascorbic acid was unable to be removed as an interfering substance due to its spontaneous conversion to oxalate.

In a related method published in 1997, HPLC was employed with an anion exchange column to resolve urinary oxalate prior to the reaction with immobilized oxalate oxidase isolated from barley [26]. The product, H_2_O_2_, was quantified using an amperometric detector. Oxalate eluted at 8.2 min, with values comparable to that of a commercial enzyme-based kit for several different concentrations. The limit of detection was 1.5 µM, which is more sensitive than the enzyme kits currently on the market (~20 µM reported). Linearity was reported up to at least 700 µM oxalate.

In 1999, urinary oxalate was measured with an alkylamine glass-bound oxalate oxidase purified from leaves of *Amaranthus spinosus* [27]. The hydrogen peroxide generated in the reaction was measured using 4-aminophenazone, phenol, and horseradish peroxidase. This reaction yielded a chromogen that absorbed 520 nm light. The results of this protocol were reportedly comparable to that of the commercially available kits. The limit of detection was 10.2 µM (0.9 µg/mL) and linearity was shown up to 300 µM. The limitations of this method, when first published, may be mitigated by improvements in biotechnology related to genetic engineering and protein purification. Some potential limitations are the use of glutaraldehyde in the immobilization process, and phenol in the detection step, which must be handled with some precautions, in addition to the 48-h reaction time for coupling oxalate oxidase to the beads. A major strength of the method is that the oxalate-oxidase-bound beads were reusable for at least 300 times after simple washing and storage in distilled water. If combined with modern technology, it appears feasible to construct a device, based on this method, for direct-to-consumer use.

In the following year, urinary oxalate was again detected after a reaction with oxalate oxidase; however, a synthetic compound, MnL(H_2_O)_2_(ClO_4_)_2_ (L = bis(2-pyridylmethyl)amino propionic acid), modeled off the catalase enzyme was thereafter reacted with hydrogen peroxide that had been generated from oxalate oxidase, forming a chromogen [29]. When absorbance of the chromogen was measured at 400 nm, the limit of detection for oxalate was found to be 2.0 µM (0.176 µg/mL). The linear range was 2.0 µM to 20 mM. It was also observed that adjusting the concentration of the catalase mimetic had a significant influence on the assay sensitivity. For example, 6 mM was required to detect the lower oxalate concentrations, whereas 1 mM was adequate for detecting 20 mM oxalate. A reported limitation of the method was that, in real urine samples, the calculated oxalate concentration was exceedingly higher than what is typically found. The investigators concluded that an additional substance (or additional substances) in urine may have reacted with the catalase mimetic to increase the absorbance at 400 nm. The identity of the interfering substance(s) was not determined. Although this limitation precludes the clinical use of the assay for oxalate quantification, revealing the limitation should lead to further exploration to determine the scope of reactivity of the catalase mimetic.

In 2003, oxalate oxidase was used with SIRE (sensors based on injection of the recognition element) technology to measure oxalate in urine [28]. In this method, oxalate oxidase was introduced into the reaction chamber by flow-injection, where it was free to react with oxalate from a sample. The hydrogen peroxide generated from the reaction was oxidized at the electrode, where the signal was then received. The advantage of this method is that oxalate oxidase is not immobilized to a support, but rather, it is used once in a small quantity before being discarded. This approach ensures that the enzyme is not degraded from thermal or other factors that can be troublesome for repeat-use biosensors. Moreover, the SIRE method allows the sample response to be easily measured in the absence of the enzyme, to correct for matrix-related interferences. The analysis time for this approach was 2–9 min, with a high selectivity imparted by oxalate oxidase, and a limit of detection of 20 µM (1.76 µg/mL). Linearity was shown up to 5 mM.

**Figure 3:**
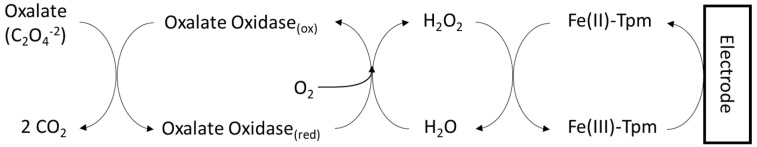
Coupling of immobilized oxalate oxidase with amperometric detection. Oxalate oxidase was immobilized on magnetic solids, which were transiently retained on an electrode. Fe(III)-tris-(2-thiopyridone) borate (Fe(III)-Tpm) was used as a mediator between enzymatically generated hydrogen peroxide and the electrode.

In 2012, a group of investigators reported coupling oxalate-oxidase-mediated oxalate metabolism with amperometric flow detection for quantifying oxalate in human urine [30]. In the study, oxalate oxidase was immobilized on a magnetic solid that was transiently retained on an electrode modified with Fe (III)-tris-(2-thiopyridone) borate. This complex served as a mediator that circumvented known issues with direct amperometric measurement of H_2_O_2_, such as interference from ascorbic acid and uric acid in urine. The reaction scheme is represented in Figure 3. The limit of detection was 11.4 µM (1.0 µg/mL) with a limit of quantification of 34.1µM. The advantages of this method are: no need for sample treatment, small sample volumes, and relatively simple equipment.

### 2.2. Liquid Chromatography–Mass Spectrometry-Based Methods

Liquid chromatography coupled with mass spectrometry (LC-MS or LC-MS/MS) has become the method of choice for many analytes in food and drug analysis. High sensitivity and unparalleled selectivity are generally a major advantage of MS; however, until recently [33,34], the limit of quantification of oxalate using MS has not realized the sensitivity advantage. For example, in 2006, urinary oxalate was quantified using a weak anion exchange chromatography column and tandem mass spectrometry with electrospray ionization in the negative ion mode [31]. The limit of detection was 3.0 µM (0.264 µg/mL), and the limit of quantification was 100 µM. Linearity was demonstrated up to 2.2 mM. This limit of quantification is higher than that of some commercially available enzyme-based kits. However, one advantage of the method was a short (1.2 min) retention time with an adequate resolution from areas of ion suppression.

In 2018, a similar method was used, wherein a solid-phase extraction plate with weak anion exchange activity was employed in resolving urine oxalate [32]. When electrospray ionization in the negative ion mode was used with tandem mass spectrometry, a limit of quantification of 60 µM was found. Linearity was demonstrated up to 1.39 mM. Both this method and the former used ^13^C_2_ oxalate as an internal standard to correct for matrix-derived ion suppression. One advantage of the method is the ability to simultaneously quantify citrate, which may be useful due to its ability to sequester calcium and thus reduce the risk of calcium oxalate stones. Some limitations of the method include a lower sensitivity than other methods, and the time required for solid-phase extraction steps, including a drying phase, and several wash phases. These disadvantages can likely be circumvented with instrumental automation.

More recently, Mu et al. reported using ion chromatography coupled with triple quadrupole MS for quantifying oxalate, and nine other organic acids, in alcoholic beverages [33]. Liquor was analyzed directly after dilution and degassing, whereas wine was purified with graphitized carbon black solid-phase extraction before analysis. Separation was achieved with a Dionex IonPac AS11-HC anion analysis column with high capacity and hydrophilicity. A gradient elution of increasing KOH strength was used to allow the increased affinity of monovalent anions (e.g., lactate) in the beginning of the separation, and decreased affinity of di- and tri-valent anions (e.g., oxalate and citrate) at the end of the separation. A desalting suppressor was used to electrolyze water and allow the exchange of K^+^ for H^+^ at a cation exchange membrane prior to the detector to avoid fouling. The total run time was 35 min, with a 26 min retention time for oxalate. Electrospray ionization tandem mass spectrometry (ESI-MS/MS) in the negative ion mode was used with multiple reaction monitoring. Oxalate showed a linear response from 0.05 to 2 mg/L (r^2^ > 0.99). Recovery of oxalate ranged from 96.5% to 107.5%, with samples subjected to solid-phase extraction (i.e., wine) showing a tendency to exceed 100%. The cause of this apparent increase in recovery was not determined, but may theoretically be related to the removal of ionization-suppressing substances during the solid-phase extraction. Advantages of the method were simple sample preparation and high sensitivity. A potential limitation is that the instrumentation is not readily available to all investigators.

Li et al. recently reported using ion-pairing reversed-phase (IP-RP) LC-MS/MS for the simultaneous quantitation of oxalate and citrate in urine and serum [34]. The method demonstrated linearity from 0.25 to 1000 µM, with a correlation coefficient > 0.99. Sample preparation was simple, involving dilution with water prior to deproteinization with methanol, then evaporation and reconstitution of the supernatant with water. ^13^C_2_ oxalate was used as a “surrogate analyte”, as a true blank matrix was not feasible. That is, oxalate and citrate could not be reliably removed from the rat serum and urine used for the method validation. Multiple ion-pairing reagents and buffers were tested for the optimization of the protocol. Recovery of oxalate from urine was 90 to 98%, while that from serum was 96 to 102%. There were no major matrix effects, with a maximum signal suppression of 7.28% found for the highest oxalate concentration. The precision was <14.70% and the accuracy was <19.73%. An advantage of the method is a shorter run time vs. other chromatography-based methods (<4 min) and better sensitivity than most reported methods to date.

Gómez et al. reported using ion chromatography with an Orbitrap mass spectrometer and electrospray ionization to quantify oxalic acid in bees, honey, beeswax, and bee bread (fermented bee pollen) [35]. The limit of detection was 0.011 µmol/kg of the sample, within-run precision was 20% relative standard deviation, and recovery was from 67% to 82%, with the lowest recovery from beeswax and highest recovery from whole bees. One potential limitation was the sample preparation time, which included disruption with an ultrasonic probe, lipid removal with a C_18_ cartridge, and deproteinization with acetonitrile. However, given the complexity of the matrices, all steps are likely necessary to avoid strong matrix effects. Indeed, a strength of the method was less than 10% signal suppression from matrices. In addition, the sensitivity was comparable to that of electrochemical methods, and the specificity of modern mass spectrometry is superior.

### 2.3. Gas-Chromatography-Based Methods

A GC-MS method was also reported in 1987 for determining urinary oxalate with ^13^C-oxalate as an internal standard [36]. This protocol required a 3 h precipitation step for oxalate with calcium, and derivatization to its isopropyl ester with propane-2-ol-HCl, and the limits of detection and quantification were not directly assessed. However, linearity was shown from 25 µL to 100 µL of 1.0 mM oxalate after drying, derivatization, and chloroform extraction.

In 1988, plasma oxalate was measured by capillary GC with flame ionization detection, and compared with GC-MS, using ^13^C-oxalate as an internal standard in the latter method [37]. In this protocol, oxalate was extracted with ethyl acetate and derivatized with MTBSTFA for 24 h. A limitation of the method, other than the reaction time, was a 57.9% oxalate recovery. Although a limit of detection was not reported, oxalate was quantified as low as 1.5 µM in plasma. This reported sensitivity is not typically achieved with most other methods discussed in this review. Lastly, the correlation coefficient between flame ionization detection and mass spectrometry was 0.938.

A recent report (2020) demonstrated that gas chromatography coupled with tandem mass spectrometry could be instrumental in measuring the oxalate synthesis rate in human subjects with or without primary hyperoxaluria (liver-generated oxalate) [38]. In this method, ^13^C-labeled oxalate and glycolate were continuously infused into human subjects to monitor tracer-to-tracee ratios (TTRs). Plasma samples were derivatized with MTBSTFA prior to GC-MS/MS analysis. The proposed major utility of the method is in measuring the efficacy of therapeutic interventions for preventing oxalate synthesis. The method was not designed to measure absolute oxalate concentrations, but rather tracer/tracee ratios. Therefore, limit of detection and quantification were not reported. However, the TTR calibration curve was linear with an R^2^ of 0.9999.

### 2.4. Electrochemical Non-Enzymatic Methods

In 2000, the oxalate content of various foods was quantified using anion exchange chromatography coupled with a conductivity measurement [16]. This method was compared with capillary electrophoresis (discussed in the next section). The anion exchange method exhibited very high sensitivity (limit of quantification of 5.68 pM) compared to other reported methods. The response was linear and oxalate recovery was complete when mechanically processed food was heated with acid. A limitation was column “poisoning”, a term used to describe contamination of the column with food substances and diminished resolution with repeated use.

**Figure 4:**
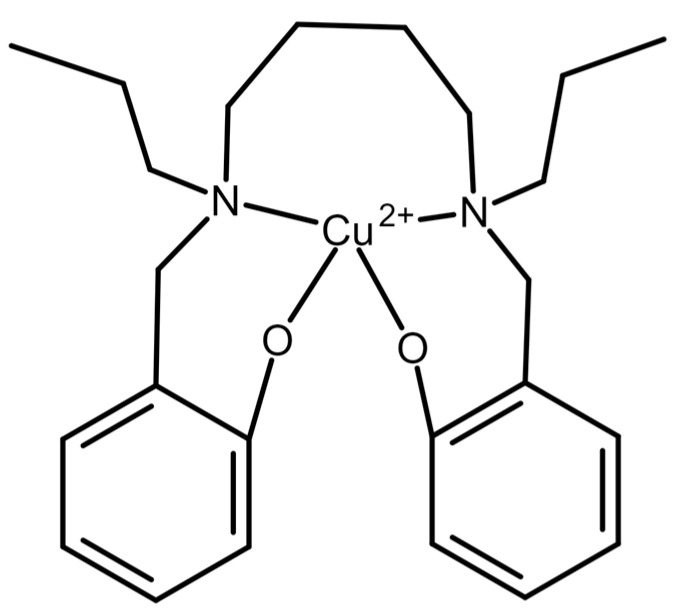
Ionophore used in [39] The ionophore, 2,2-[1,4-butandiyle bis(nitrilo propylidine)]bis-1-naphtholato copper(II) was used in a PVC-based ion-selective electrode for detection of oxalate.

In 2006, an oxalate-selective membrane electrode based on polyvinyl chloride and the complex and ionophore, 2,2′-[1,4-butandiyle bis(nitrilo propylidine)]bis-1-naphtholato copper(II) (Figure 4), was used to quantify oxalate in water [39]. The limit of detection was 0.05 µM (4.4 × 10^−3^ µg/mL), and the linear range was 0.05 µM to 1.0 × 10^5^ µM. Noted advantages, other than the sensitivity and dynamic range, were the fast response time of 10–15 s, and the ability to use the same electrode for 3 months. In addition, the electrode was several orders of magnitude more selective for oxalate than other anions, except for perchlorate and OH^−^ (when tested in alkaline conditions). However, the utility of the method in a more complex sample matrix, such as urine or plasma, is unknown. If refined to maximize selectivity and proven to be suitable in complex fluids, this method could potentially be preferred due to its minimal need for sample handling or complex equipment.

In 2009, a method for the sensitive and selective detection of oxalate in aerosols using microchip electrophoresis was reported [40]. In this study, the investigators used micellar electrokinetic chromatography (MEKC), which takes advantage of the ability of surfactant micelles to modify separations of weakly solvated anions. Using this approach, oxalate, being a highly solvated anion, was readily separated from the micelle-influenced analytes. In addition, picolinic acid was used in order to chelate metals that would otherwise bind and reduce the detector response to oxalate. Detection was achieved with a Dionex conductivity detector, and the limit of detection for oxalate was 180 nM (1.67 × 10^−3^ µg/mL), with a 19 nM limit when field-amplified sample stacking was used. This sensitivity is a major advantage of the method, exceeding that of a large majority of other methods reported to date. In addition, a major advantage of this method is the ability for the continuous online monitoring of the aerosol composition due to the sample run times of less than 1 min. Disadvantages of this method include a loss of response linearity above 300 µM, poor resolution between oxalate and the internal standard at higher concentrations, and a change in bulk solution conductivity with higher analyte concentrations. The latter limitation needs to be corrected by internal standard addition. Finally, this method has not been tested in biological samples, such as urine or plasma.

In 2022, Moura et al. used ion chromatography with a conductivity detector to quantify oxalate and other anions (and cations) in *C. elegans* [41]. Worms were digested with 35% H_2_O_2_ and microwave radiation, then dried, resuspended in water, and filtered. The filtrate was used directly for analysis. Separation was performed on a DIONEX IonPac AS19 analytical column with AG19 guard column. A gradient elution was used with a KOH gradient with a suppression system. A dual-channel conductivity detector was used, one channel for anions, the other for cations. The method showed linearity from 1.0 to 100 µg/L (r^2^ = 0.9999), with a limit of detection of 0.273 µg/L and a limit of quantitation of 0.827 µg/L.

In 2023, Kotani et al. used semi-micro hydrophilic interaction liquid chromatography coupled with electrochemical detection (HILIC-ECD) to quantify oxalate in herbal products used in traditional Kampo medicine [42]. Kampo is the study of traditional Chinese medicine in Japan. Herbal products evaluated in this study included Zingiberis Rhizoma Processum, Pinelliae Tuber, Sho-seiryu-to, Hange-shashin-to, Kami-shoyo-san, Bakumondo-to, and Daikenchu-to. Preparation of samples involved heating in a 10% *w*/*v* HCl solution at 90 °C, centrifugation, dilution of supernatant in 10% *w*/*v* HCl, then dilution in acetonitrile/phosphate buffer prior to filtration through a PTFE filter. An Intersil Amide column (250 × 1.0 mm i.d., 3 µm) was used for separation at 50 °C, and detection was at an applied potential of +1.1 V vs. Ag/AgCl. This method demonstrated a broad linear range, from 4.5 × 10^−4^ to 1.8 µg/mL (0.0019 µM limit of detection), making it one of the most sensitive methods reported to date. A limitation was that the oxalic acid peak appeared to approach the limit of detection in Sho-seiryu-to extracts.

### 2.5. Indicator-Displacement-Based UV, Colorimetric, and Fluorescence Methods

**Figure 5:**
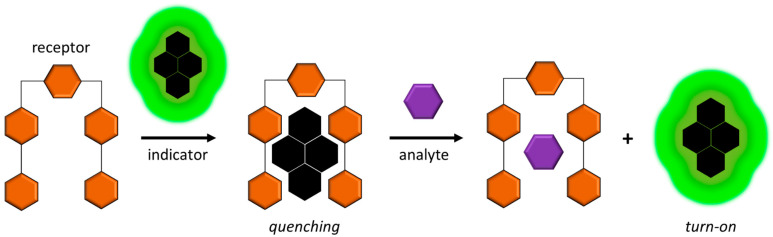
Principle of operation of indicator-displacement-based fluorescent chemosensors. The receptor (e.g., macrocyclic metal complex) sequesters the indicator, inhibiting fluorescence. The analyte of interest is added to displace the indicator, regaining fluorescence.

There is substantial interest in developing indicator-displacement-based methods for detecting oxalate that do not require enzymes, liquid chromatography, or mass spectrometry. Many of these methods quantify fluorescence quenching, or, more often, gain of fluorescence upon addition of the analyte [54]. The principle of operation is shown in Figure 5. A capillary electrophoresis method coupled with indicator displacement and UV absorbance [16] and a colorimetric method [49] have also been reported. These approaches are briefly discussed here.

In 2000, the oxalate content of foods was estimated via capillary electrophoresis coupled with the UV absorbance (254 nm) response to oxalate-mediated chromate displacement [16]. The reported limit of quantification was 5.68 pM, which is the greatest sensitivity that we have found reported to date. Limitations included the need for adjustment of the electrolyte concentration for samples with low oxalate concentrations, and heating samples for 1 h. There is also a potential for oxalate to co-elute with interfering compounds, but this limitation was not observed in the study.

In 2018, a colorimetric method was developed wherein oxalate inhibited the reaction of curcumin nanoparticles with Fe (III) in acidic media [49]. The absorbance of 427 nm light by the nanoparticles showed a linear increase proportional to the oxalate concentration from 0.15 to 1.70 µg/mL. The limit of detection was 0.87 µM (0.077 µg/mL). The method was demonstrated to work well for the quantification of oxalate in water, food, and urine samples. Advantages of the method include simplicity and sensitivity. A potential limitation is interference from other substances, which was demonstrated for copper and carbonate/bicarbonate, and was not assessed for other common anions in urine, such as citrate and urate. Indeed, all indicator displacement methods are limited by either known interferences, or untested putative interferences.

A recent report by Hontz et al. revisited a fluorescence-based indicator displacement method, clearly demonstrating that interference from untested compounds in biological samples may be more prevalent than is sometimes suggested [54]. Specifically, it was shown that a dinuclear copper(II)-based macrocycle with the ability to quench eosin Y fluorescence, although reported as oxalate-selective, exhibited a stronger response to citrate and oxaloacetate. Other copper-based oxalate sensors, also based on reversing quenching, have been reported [55,56]. The reported sensitivities of these methods are in the low µM range; however, we have found that all studies to date have not performed a comprehensive assessment of potentially interfering compounds. For example, Rhaman et al. reported a nickel-based macrocycle that bound to oxalate in water [57]. Oxalate binding to the macrocycle triggered a red shift in eosin Y fluorescence with a corresponding visible color change. Potential interfering ions tested in this study included F^–^, Cl^–^, Br^–^, I^–^, NO_3_^–^, ClO_4_^–^, SO_4_^2–^, and PO_4_^3–^. Although none of the ions demonstrated interference, organic anions (e.g., citrate, urate, or oxaloacetate), were not tested. In contrast to reversing fluorescence quenching, at least one investigation reported the reversal of a fluorescence “turn on” response [58]. Specifically, Tang et al. showed that Zn^2+^ could turn on the fluorescence of a binaphthol-quinolone Schiff base. This gain in fluorescence could then be reversed via oxalate-mediated chelation and displacement of the zinc ion. In this study, phthalate, isophthalate, terephthalate, succinate, glutarate, adipate, and malonate showed no significant interference with the assay when tested at an equimolar concentration to oxalate; however, other anions were not tested, and it remains unknown whether high concentrations of the tested anions would interfere.

In 2020, Chen et al. reported the detection of oxalate via a selective recognition reaction using cadmium telluride (CdTe) quantum dots [51]. In this approach, the reduction of Cu^2+^ to Cu^+^ by oxalate was measured by quantifying the change in the fluorescence intensity of CdTe quantum dots using an excitation wavelength of 365 nm, and an emission wavelength range of 570 to 720 nm. Cu^+^ was able to quench the fluorescence of the CdTe quantum dots much more effectively than Cu^2+^. Therefore, as the concentration of oxalate increased, fluorescence decreased proportionately. This method was successfully employed with clinical urine samples, even in the presence of hematuria (presence of blood). In this study, the specificity of the assay for oxalate was measured by comparing against NaCl, KCl, MgCl_2_, Na_2_HPO_4_, NaH_2_PO_4_, urea, and glucose. No interference was observed; however, organic anions (e.g., citrate) were not assessed for interference. With optimization, the investigators achieved a linear response to oxalate from 100 nM to 10 mM in urine, with a limit of detection of 80 nM. Recovery of added oxalate was 96 to 105%. The method was able to differentiate between uric acid nephrolithiasis and calcium oxalate nephrolithiasis in clinical samples. When tested against an LC-MS method, the CdTe-based method gave similar results; however, repeatability was not assessed, and a correlation coefficient was not calculated. A more recent advancement was reported by Chen et al. using CdTe quantum dots, wherein printed test strips were able to detect oxalate ranging from 10 pM to 10 nM within 3 min [59].

More recently (2023), Wu et al. reported using Fe(III)-sulfosalicylate as a colorimetric chemosensor of oxalic acid in model wastewater undergoing photocatalytic ozonation [53]. In this method, oxalic acid stoichiometrically removes Fe(III) from Fe(III)-sulfosuccinate, producing a colorless product measured by spectrophotometry. The linear range of the assay was 0.80–160 mg/L (r^2^ = 0.9993), with a limit of detection of 0.74 mg/L. Results of the assay were comparable to those of an HPLC method. The sensor was synthesized using a simple process involving mixing an aqueous solution of FeCl_3_ with a solution of 5-sulfosalicylate, and diluting with a 0.01 M HCl solution (final pH of 1.9). Oxalic acid in model wastewater mixed with P25 TiO_2_ and simulated sunlight (AM1.5) was used with continuous ozone bubbling. To quantify oxalic acid, the catalyst was removed, the solution was mixed with the sensor solution, then the absorbance at 505 nm was measured using a standard UV-Vis spectrophotometer. A limitation identified in the study was significant interference from other dicarboxylic acids, such as methylmalonic acid, malonic acid, and succinic acid. Moreover, the assay was tested only in model wastewater.

Several additional colorimetric or fluorescent indicator-based methods have been reported, generally with lower sensitivity than electrochemical or mass-spectrometry-based methods [43,44,45,46,47,48,50,52]. All of these methods have the advantage of simple sample preparation, but invariably, they have either not been tested for interference from other organic anions, or interference has clearly been demonstrated. Given the proliferation of such studies from 2000 to 2023, there is a palpable need to improve data interpretation and extrapolation to avoid false conclusions of high specificity.

### 2.6. Summary of Analytical Methods for Quantifying Oxalate

**Figure 6:**
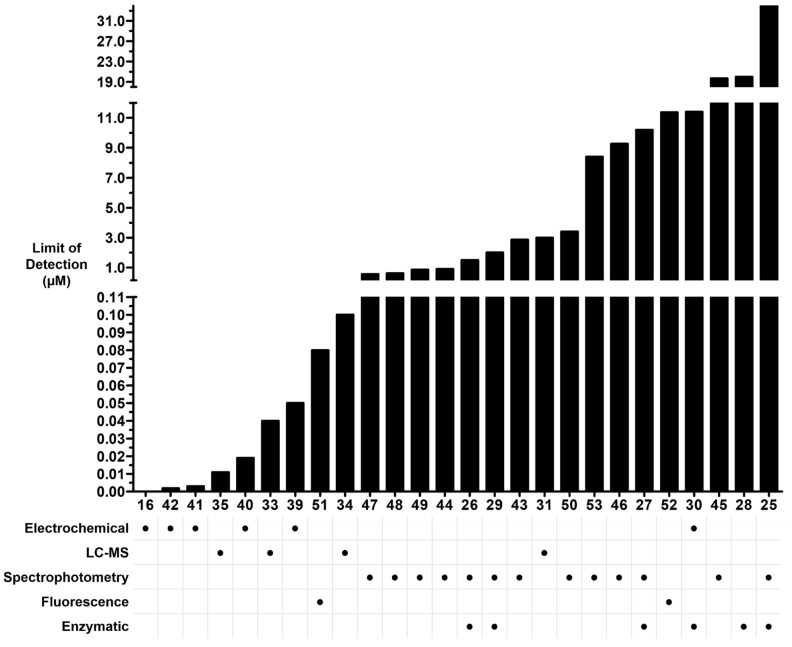
Limit of detection (LOD) of oxalate vs. mode of detection. Bars are broken for visibility of lower LOD. Numbers on horizontal axis represent reference numbers. LOD for reference 16 was reported at 0.2 mg/100 g in food, but linearity in final solution started at 5.68 × 10^−6^.

In summary, recent studies using LC-MS/MS-based methods have demonstrated a potential for high sensitivity in detecting oxalate (Figure 6). Although the sensitivity of GC-MS can theoretically be superior to several other methods, this has not been realized to date for oxalate. Moreover, the requisite derivatization step to volatilize oxalate makes sample preparation more time-consuming for GC-MS. Electrochemical methods have shown the greatest sensitivity, but LC-MS/MS is nearly eclipsing such approaches (Figure 6). Limitations of electrochemical detection include interference from variation in bulk solution conductivity, column fouling, and poor resolution for methods using column- or capillary-based modes of separation. Non-enzymatic fluorescent or colorimetric indicator methods have realized a high sensitivity and relative simplicity of instrumentation and reagents. A major limitation to these methods is the lack of specificity, or untested specificity, against common organic anions in the matrices tested.

## 3. Conclusions and Future Perspective

While oxalate plays a crucial role in various biological and industrial applications, it can be a burden for a considerable portion of the population. Its involvement in diseases, drug- and toxin-induced disorders, and disruption of industrial processes makes it a high-priority chemical for the development of more precise, selective, and straightforward analytical methods. Enzyme-based colorimetric methods have been a mainstay in oxalate analysis for many years, but the relatively low sensitivity, high cost, and significant time investment in sample preparation warranted the development of alternative methods.

Currently, there are no universally applicable, or superior, methods. Advances in mass spectrometry, coupled with improvements in orthologous separation methods for oxalate, has led to a more desirable sensitivity and specificity. The future utility of alternative methods depends on whether improvements in specificity are achieved, specifically for indicator-based methods. We propose that our review may serve as a valuable guide for researchers to select the most suitable methodology based on their objectives and instrumental context.

## Figures and Tables

**Table 1:** Methods used for detection and quantification of oxalate. LOD, limit of detection; LOQ, limit of quantification; NR, not reported; RSD, relative standard deviation; CV, coefficient of variation.

Method Type	LOD µM	LOD µg/mL	LOQ µM	Other Figures of Merit	Sample Preparation	Summary	Strengths	Limitations	Ref. Year
Enzymatic—flow injection analysis	34.0	2.99	NR, but linearity was shown from 5 to 50 µM	Recovery mean: >85% Precision not reported. Accuracy vs. other methods not reported.	Urine samples were acidified with HCl to pH 2–3 for storage, then diluted 10 × with citrate buffer (pH 3.5) prior to analysis.	Flow injection analysis (FIA) was used to measure oxalate in urine. A column reactor of immobilized oxalate oxidase was used. Generated H_2_O_2_ was measured by chemiluminescence via a reaction of luminol with hexacyanoferrate(III). L-ascorbic acid interference with the assay was unable to be eliminated with the use of sodium nitrite.	Highly specific for oxalate.	L-ascorbic acid interference was a potential problem, but was avoided by diluting the urine samples.	[25] 1994
Enzymatic—with HPLC-UV	1.5	0.13	NR, but linearity was shown up to 700 µM	Within-run precision: ≤6.7% CV Between-batch precision: ≤8.6% CV Recovery mean: 102% (10.5% CV) Accuracy: reported as statistically equivalent to commercial enzyme kit (no r value provided)	24 h urine samples were preserved with 5 mL 5% thymol-isopropanol. Blood was heparinized, centrifuged to obtain plasma, then plasma deproteinized (2 mL plasma, 40 µL concentrated HCl). Precipitated protein was removed by centrifugation. Supernatant was used directly for HPLC.	An anion exchange column was used prior to reaction of oxalate with barley oxalate oxidase in the flow path. Product, H_2_O_2_, was measured with an amperometric detector.	Oxalate was resolved in 8.2 min, with comparable accuracy to commercial enzyme-based kits. Higher sensitivity than enzyme-based kits.	Requirement for immobilization of oxalate oxidase.	[26] 1997
Enzymatic/Spectrophotometric—alkylamine glass-bound oxalate oxidase measured by a color reagent	10.2	0.9	NR, but linearity was shown up to 300 µM	Within-run precision: ≤4.0% CV Between-batch precision: ≤3.0% CV Recovery mean: ~98% Accuracy: r = 0.98 vs. commercial enzyme kit	24 h urine samples were acidified with HCl, then pH adjusted to 5–7 using NaOH. Urine was diluted 1:1 with 0.1 M potassium phosphate buffer (pH 7.0). Sodium nitrite (35 mg/10 mL) was added to avoid ascorbate interference.	Urinary oxalate was measured with an alkylamine glass-bound oxalate oxidase purified from leaves of Amaranthus spinosus. H_2_O_2_ is generated from urinary oxalate, which is measured with a colored reagent containing 4-aminophenazone, phenol, and horseradish peroxidase. The chromogen formed is read at 520 nm. Recovery of oxalate was 98%. Results were comparable to the Sigma kit method (r = 0.98).	Oxalate-oxidase beads were reusable up to at least 300 times.	48 h reaction time required. Use of hazardous reagents (glutaraldehyde and phenol).	[27] 1999
Enzymatic—oxalate-oxidase-based biosensor	20.0	1.76	NR, but linearity was shown up to 5 mM	Within-run precision: ≤6.5% RSD. Between-batch precision: ≤12% RSD. Recovery: not reported Accuracy: r = 0.952 vs. colorimetric method	EDTA was mixed with urine to 10 mM. pH was adjusted to 5.0 with succinic acid, then samples were diluted with equal volume of diluent (succinic acid and NaOH to pH 5.0). Diluted urine was mixed with 200 g/L activated charcoal, filtered, and analyzed.	Oxalate oxidase was used with SIRE (sensors based on injection of the recognition element) technology. Analysis was selective, simple, and inexpensive. Linear between 0–5 mM or more with a shorter reaction time. Results were comparable to a colorimetric method.	Minimal enzyme degradation and correction for matrix-related interferences. Rapid analysis time (2–9 min)	Limit of detection relatively high.	[28] 2003
Enzymatic—catalase model compound with oxalate oxidase quantified by UV-vis	2.0	0.176	0.5 Linear from 0.5 to 25 µM (R = 0.996)	Within-run precision: ≤4% CV Recovery mean: ~96% Accuracy: mean oxalate concentration was ~95% of that with enzyme method (no regression was performed vs. enzyme method)	Urine samples treated with HCl to pH 1–2. EDTA was added to 10 mM to prevent calcium oxalate precipitation. pH was adjusted to 5 with succinic acid, diluted, then mixed with activated charcoal before filtration and storage at −20 °C.	Urinary oxalate was determined based on a catalase model compound MnL(H_2_O)_2_(ClO_4_)_2_ (L = bis(2-pyridylmethyl)amino)propionic acid). Oxalate is decomposed with oxalate oxidase into CO_2_ and H_2_O_2_. H_2_O_2_ is converted to H_2_O and O_2_ by the catalase model compound, forming a color compound. Absorbance of the formed compound is measured. Linear from 0.002–20 mM oxalate. Results are consistent with enzymatic and HPLC methods. The compound modeled off catalase avoids the need for enzymes.	High sensitivity.	Real urine samples yielded a higher oxalate concentration than expected.	[29] 2009
Enzymatic/Electrochemical—amperometric biosensor used on urine	11.4	1.0	34.1 Linear from 3.0 to 50.0 mg/L (r^2^ = 0.999)	Within-run precision: ≤2.0% RSD. Between-batch precision: ≤2.2% RSD. Recovery: not reported Accuracy: not significantly different from spectrophotometric detection.	Urine was mixed with a saturated CaCl_2_ solution, centrifuged to collect precipitate. Precipitate was dissolved in H_2_SO_4_ 0.1 M for direct analysis.	Oxalate oxidase enzyme was immobilized on a magnetic solid containing an electrode modified with Fe (III)-tris-(2-thiopyridone) borate. Oxalate quantification was linear between 3.0–50.0 mg∙L^−1^.	No sample treatment, small volume, and simple instrumentation.	Relatively high limit of quantification.	[30] 2012
LC-MS/MS and anion exchange chromatography	3.0	0.264	100.0 Linear from 100.0 to 2212 µM (R^2^ = 0.999)	Within-run precision: ≤4.0% CV Between-batch precision: ≤5.0% CV Recovery mean: 95% Accuracy: r = 0.964 vs. enzymatic assay	Urine samples (10 µL) were added to 10 µL internal standard (^13^C_2_-oxalic acid) solution and 400 µL water for injection from a microtiter plate.	Urinary oxalate was quantified using weak anion exchange chromatography and MS/MS. Used a Waters OASIS WAX column, and detected oxalate 88.6 > 60.5 m/z transition and a 90.5 > 61.5 transition for 13C2-oxalate. Electrospray negative ion mode was used. Oxalate eluted at 1.2 min, 95% recovery; LOQ was 100 umol/L. Linear to 2212 umol/L. Oxalate eluted away from area of ion suppression. No interference from organic acids. Quantitative results were comparable to enzyme assay	Retention time afforded resolution from areas of ion suppression. Short (1.2 min) retention time.	Relatively high limit of quantification.	[31] 2006
LC-MS/MS and WAX solid-phase extraction	NR	NR	60.0 Linear from 60 to 1388 µM (R^2^ > 0.99)	Within-run precision: ≤11.1% CV Between-batch precision: ≤5.4% CV Recovery mean: 100% Accuracy: individual samples were −34% to +74.2% different from enzymatic method (attributed to ion suppression by sample matrix)	Urine was pipetted into 96-well plate, internal standard (pH 1.6 in PBS) was added prior to 2% formic acid. Plate was mixed before well contents were pushed through a methanol/water pre-treated WAX-SPE plate, followed by several washes. Flow-through was discarded. Contents were eluted with ammonia in methanol, evaporated, dissolved in 100 µL water, centrifuged, and injected directly from plate.	Measured urine citrate and oxalate after a simple weak anion exchange solid-phase extraction (WAX-SPE) clean-up procedure. Detected with electrospray-negative ion mode. Linear from 60 to 1388 umol/L.	Reaction monitoring of oxalate 88.9 > 60.85 allows for more confident identification. Oxalate eluted at 0.29 min. 100% oxalate recovery.	Manual SPE plate loading, eluent evaporation, and reconstitution. Required ^13^C use as an internal standard to correct for matrix-derived ion suppression.	[32] 2018
Ion chromatography-MS/MS and solid-phase extraction	0.040	0.0035	0.137	Within-run precision: 4.7 to 6.2% RSD Recovery: 96.5% to 107.5% Accuracy: not compared to other methods, but spiking and recovery was performed.	Liquor or wine was purified through solid-phase extraction and used directly for analysis.	Samples were separated on a Dionex IonPac AS11-HC anion analysis column with increasing KOH strength in eluent. K^+^ was removed with an in-line cation exchange-based suppressor prior to ESI-MS/MS in negative ion mode.	Simple sample preparation. High sensitivity.	Specialized instrumentation and technical skill. Separation time may be a limitation compared to indicator-based multi-well plate methods.	[33] 2022
Ion-pairing reversed-phase LC-MS/MS	0.10	0.0088	0.25 Linear from 0.25 to 1000 µM (R^2^ > 0.99)	Intra-day precision: ≤5.82% Inter-day precision: ≤10.32% RSD Recovery: 90% to 102% Accuracy: ≤17.38% Matrix effect: max signal suppression was 7.28%.	Urine or serum was diluted with water, deproteinized with methanol, supernatant evaporated, and reconstituted with water containing ^13^C_2_ oxalate as a standard.	An Agilent 1290 UHPLC LC system was coupled to an Agilent 6490 triple quadrupole MS system with electrospray ionization. Ion-pairing reversed-phase (IP-RP) LC-MS/MS was used for simultaneous citrate and oxalate quantitation. A Waters Acquity UPLC BEH C18 column used with an ion-pairing buffer containing 5 mM N,N-dimethylcyclohexylamine (DMCHA) and 25 mM hexafluoro-2-isopropanol (HFIP).	Simple sample preparation. High sensitivity. Short retention times (<4 min run time).	Specialized instrumentation and technical skill.	[34] 2022
Ion chromatography with quadrupole Orbitrap mass analyzer	0.011 µmol/kg	0.001 mg/kg	20 mg/kg Linear from 20 to 1000 mg/kg (R^2^ = 0.999)	Within-run precision: <20% RSD Recovery: 67% (beeswax) to 82% (bee) Matrix suppression: <10% for beeswax, and enhancement for other samples Accuracy: not compared to other methods	Bees disrupted via ultrasonic probe extraction in water, then dispersive solid-phase extraction. Filtrate was added to a tube with Bondesil-C_18_ (lipid removal) and acetonitrile (deproteinization), vortexed, centrifuged, and supernatant collected. Honey was diluted with water, heated to 35 °C, and agitated. It was then centrifuged, and supernatant collected. Bee bread (fermented pollen) or beeswax was added to water, vortexed, shaken, then centrifuged. Supernatant was mixed with Bondesil-C_18_ and acetonitrile, vortexed, centrifuged, and supernatant collected.	A Thermo Scientific^TM^ Dionex^TM^ Integrion^TM^ HPIC^TM^ system was used with water and an increasing KOH gradient as mobile phase. An AS11-HC column was used for separation. An AERS 500es 2 mm suppressor was used to remove K^+^. A Thermo Scientific^TM^ Orbitrap Exploris^TM^ 240 mass spectrometer with an OptaMax^TM^ NG (H-ESI II) ion source was used. Oxalic acid was analyzed with simultaneous MS and targeted MS^2^ modes. The retention time of oxalic acid was ~12.25 min.	Sensitive. Specific. Low matrix effect.	Poor recovery. Specialized instrumentation and training required.	[35] 2022
GC-MS	NR, but 25 nmol mass was reported as detectable			Within-run precision: ≤2.6% CV Between-batch precision: ≤3.0% CV Recovery mean: 103–109% Accuracy: r = 0.919 vs. enzyme assay kit, r = 0.9906 vs. HPLC.	Urine was acidified with HCl to pH < 3 and stored at −20 °C until analysis. 100 µL urine was mixed with 50 µL of a 1 mM [^13^C]oxalic acid solution. 0.5 mL saturated CaSO_4_ solution and 3 mL absolute ethanol was added. Samples were mixed and allowed to stand for 3 h. Precipitate was centrifuged, supernatant discarded, and precipitate was dried under N_2_ prior to adding 75 µL of propane-2-ol-HCl and incubating for 75 min at 80 °C. Then, 1 mL each, chloroform and water, were added. Samples were vortexed, centrifuged, and aqueous layer was collected. The wash was repeated twice. 1 µL of the organic later was injected for GC-MS.	Used a stable isotope (^13^C) of oxalate as an internal standard added to urine. Oxalate was converted to its isopropyl ester using propane-2-ol-HCl.	N/A	Requires a 3 h precipitation step and drying step. Uses chloroform.	[36] 1987
Capillary GC-flame ionization vs. GC-MS	NR, but oxalate was quantified as low as 1.5 µM			Between-batch precision: CV was ≤15.9% for GC and ≤14.9% for GC-MS Recovery mean: 57.9% Accuracy: r = 0.938 (GC-flame ionization vs. GC-MS)	0.5 mL plasma was mixed with 0.25 g NaCl and 250 µL 0.1 M HCl. Samples were then extracted twice with 2 mL ethyl acetate. Ethyl acetate fractions were dried over anhydrous sodium carbonate. Malonic acid was added as an internal standard for GC-flame ionization. Sodium [^13^C_2_]oxalate was added as an internal standard for the GC-MS method. Then, 1.5 mL portions were evaporated to ~50 µL under nitrogen prior to derivatization with 30 µL MBSTFA at 50 °C for 30 min.	Plasma oxalate was extracted and derivatized with MTBSTFA prior to capillary gas chromatography and flame ionization. GC-MS showed a 0.938 correlation coefficient with the method, wherein ^13^C-oxalate was used as an internal standard.	Reported quantifying oxalate as low as 1.5 µM.	24 h derivatization step, and a 57.9% oxalate recovery when extracted with ethyl acetate.	[37] 1988
GC-MS/MS	NR			Within-run precision: ≤4.2% CV Between-batch precision: ≤6.3% CV Recovery mean: not determined Accuracy: absolute oxalate concentration not determined	200 µL plasma samples were mixed with 30 µL 12 M HCl. Then, 50 µL 100 mg/mL ethoxylamine solution in water, and 100 µL water were added. Mixtures were incubated at 80 °C for 30 min before 50 µL NaCl-saturated water was added. 1 mL ethyl acetate was added before vortexing and centrifuging. The upper later was transferred to a GC vial. The extraction was repeated and combined. Samples were evaporated before adding 25 µL MBSTFA and 25 µL acetonitrile. The mixture was incubated for 30 min at 80 °C, cooled, and analyzed.	Stable isotopes (^13^C) of oxalate and glycolate were continuously infused into human subjects to monitor tracer-to-tracee ratios (hepatic metabolism). Plasma samples were derivatized with MTBSTFA and analyzed by GC-MS/MS.	Ideal for determining efficacy of therapeutic interventions for treating primary hyperoxaluria (hepatic oxalate synthesis).	Not designed for quantifying absolute oxalate concentrations.	[38] 2020
Electrochemical—anion exchange column chromatography with conductivity detection	NR	NR	0.2 mg/100 g in food Linear from 5.68 × 10^−6^ to 1.14 × 10^−4^ µM (r^2^ = 0.99)	Within-run precision: 0.8% to 4.0% CV depending on the food. Recovery mean: 98–100% Accuracy: r = 0.99 vs. capillary electrophoresis	2–3 g food was chopped and homogenized with 9 volumes of 0.2 M HCl. Homogenates were incubated at 60 °C for 1 h prior to centrifugation. Supernatants were filtered through a 0.2 µm pore PTFE filter. Samples were then diluted 50–500 x with water prior to analysis.	Anion exchange chromatography with conductivity detection was shown to be superior to capillary electrophoresis for estimating low oxalate concentrations.	Very high sensitivity. Complete oxalate recovery. Linear response.	Retention time of oxalate was up to 15.2 min. Column “poisoning” was reported, wherein food components altered column chemistry over time. Oxalate appeared to elute on a broader peak (not baseline resolved) in apple extract. However, this was not observed in foods of higher oxalate content.	[16] 2000
Electrochemical—oxalate-selective membrane electrode	0.05	4.4 × 10^−3^	0.05	Not reported	Oxalate was prepared in water.	Used 2,2′-[1,4-butandiyle bis(nitrilo propylidine)]bis-1-naphtholato copper(II). Linear detection of oxalate from 0.05 to 100,000 µM	Sensitive. Fast response time (10–15 s) and could be used for more than 3 months.	Only used in water samples.	[39] 2006
Electrochemical—ion chromatography and microchip electrophoresis	0.180, down to 0.019 with field-amplified sample stacking.	1.67 × 10^−3^	0.04 Linear from 0.04 to 300 µM (r^2^ = 0.9997)	Precision not reported. No comparison was made to determine accuracy against other methods.	Aerosols from air samples were collected in an outdoor mountainous region using an automated annular denuder/filter pack system. Denuders coated in sodium chloride or phosphorous acid, in series, were used to remove gaseous HNO_3_, SO_2_, and NH_3_. A filter pack with nylon filter was used to collect fine particles. The filter was extracted with water and sonication. Extracts were used in ion chromatography or microchip capillary electrophoresis with a conductivity detector.	Used a zwitterionic surfactant with affinity for solvated anions (N-tetradecyl,N,N-dimethyl-3-ammonio-1-propanesulfonate). Separation was performed at pH 4.7, permitting pH manipulation of oxalate’s mobility.	Sensitive. 1 min separation. Allows continuous online monitoring of aerosol composition.	Tested with atmospheric aerosols, but not biological samples. Poor resolution of oxalate and the internal standard at higher concentrations. Loss of linearity above 300 µM oxalate. Change in bulk solution conductivity with higher analyte concentrations, requiring internal standard-based correction.	[40] 2009
Electrochemical—ion chromatography with conductivity detection	0.003	2.729 × 10^−4^	0.009 Linear from 0.011 to 1.14 µM (R^2^ = 0.9999)	Within-run precision: ≤5.68% RSD Between-batch precision: ≤0.97% RSD Recovery: 71–83% Accuracy: not compared to alternative methods Matrix effect: method was not susceptible to matrix effect	*C. elegans* worms were digested with 35% H_2_O_2_ and microwave radiation, dried, resuspended in water, and filtered.	Ion chromatography using a Dionex™ Inegrion™ HPIC™ System with a Dionex IonPac AS19 column was performed, and detection was with double-channel conductivity detector to quantify anions or cations. Gradient elution with increasing KOH was used for oxalate and other anions. Pre-detector K^+^ suppression was achieved with an in-line suppressor (AERS-500 2 mm)	Highest sensitivity of all methods. Simple sample preparation.	~30 min run time. Not amenable to high-throughput analysis. Specialized instrumentation and technical skill.	[41] 2022
Electrochemical—semi-micro hydrophilic LC (HILIC) with electrochemical detection	0.0019	1.7 × 10–4	Linear from 4.5 × 10^−4^ to 1.8 µg/mL (r = 0.999)	Within-run precision: 0.3% to 2.9% RSD depending on herbal product Recovery: 88.7% to 104.2%	Crude herbal medicines were heated in HCl solution for 30 min at 90 °C centrifuged, supernatant diluted with HCl solution, then further diluted with acetonitrile-phosphate buffer (65:35, *v*/*v*). Sample was filtered through 0.45 µM PTFE filter, and 5 µL loaded into HILIC-ECD system.	Hydrophilic interaction liquid chromatography coupled with electrochemical detection (HILIC–ECD) was used. An Intersil Amide column was used at 50 °C. Retention time for oxalic acid was 8.1 min. Applied potential was +1.1 V vs. Ag/AgCl.	High sensitivity. Simple sample preparation.	Without mass spectrometry, cannot confirm that peak was pure oxalic acid. However, shape did not indicate any impurities.	[42] 2023
Spectrophotometric—capillary electrophoresis coupled with 254 nm UV absorbance	NR	NR	0.2 mg/100 g in food Linear from 5.68 × 10^−6^ to 1.14 × 10^−4^ µM (r^2^ = 0.99)	Within-run precision: 9.4% to 26.8% CV depending on the food. Recovery mean: 99–114% depending on the food Accuracy: r^2^ = 0.99 vs. ion chromatography	2–3 g food was chopped and homogenized with 9 volumes of 0.2 M HCl. Homogenates were incubated at 60 °C for 1 h prior to centrifugation. Supernatants were filtered through a 0.2 µm pore PTFE filter. Samples were then diluted 50–500 × with water prior to analysis.	Various foods were processed and heated to extract oxalate. Oxalate was resolved using capillary electrophoresis, and quantified by measuring change in UV absorbance of oxalate-displaced chromate.	Very high sensitivity. Complete oxalate recovery. Linear response.	Less specific compared to mass spectrometry or enzyme-based methods. Electrolyte concentration required adjustment for oxalate concentration. Sample heating for 1 h required. Poor precision compared to anion exchange chromatography.	[16] 2000
Spectrophotometric—oxalic acid-mediated catalysis of rhodamine B fading	2.87	0.252	Linear from 0.40 to 6.0 µg/mL (γ = 0.999)	Within-run precision: 2.4% RSD Recovery: 95.8% to 102.8%	Tea leaves were added to 100 °C water, soaked, and filtered. Spinach was boiled in water for 1 h, then filtered. Urine was diluted and used directly.	Sulfuric acid, rhodamine B, and potassium dichromate were mixed in an aqueous solution. Oxalic acid-containing samples were added, and the reaction was heated at 50 °C for 10 min. After cooling, NaOH was added, then absorbance measured at 552 nm to monitor oxidation of rhodamine B by oxalic acid.	Reaction can be easily terminated with NaOH. Absorbance stable for >3 h.	Interference from several inorganic ions causing >5% error at concentrations lower than oxalate.	[43] 2006
Spectrophotometric—oxalate activation of Fe(II) oxidation of iodide by bromate	0.91	0.08	Linear from 0.10 to 7.0 µg/mL (r = 0.9989), but data not shown.	Within-run precision: 1.8% to 4.0% RSD Recovery from spiked water: 94.5% to 103% Accuracy: was compared to reference method, but details on reference method not provided.	Spinach or mushroom was pulverized in a mortar, mixed with water, and boiled for 20 min prior to filtration.	Oxalate-containing solutions were mixed with acetate buffer (pH 5.0) and Fe(II) and potassium iodide. Sodium bromate was added, and solutions were read in a spectrophotometer at 352 nm absorbance, which increased over time proportionally with the oxalate concentration.	Simple sample preparation.	Interference from divalent and trivalent cations via precipitation with oxalate. Overcome by acidification and cation exchange resin. Not tested against other divalent organic anions.	[44] 2006
Spectrophotometric—oxalic acid replacement of of dibromochloroarsenazo in Zr(IV) complex	19.7	1.73	Linear from 50 to 300 µM (r = 0.9994)	Within-run precision: 0.24% to 1.78% RSD depending on tea type Recovery: 98.8% to 102% Accuracy: results were comparable to method in [43]	Tea leaf was soaked with boiled water for 30 min, and filtered.	An HCl solution, zirconium solution (ZrOCl_2_·8H_2_O), and DBC-arsenazo (C_22_H_14_AsBr_2_ClN_4_O_11_S_2_) solution, were mixed prior to addition of oxalic acid-containing samples. After 20 min incubation, absorbance was read at 500 nm.	Simple sample preparation	Low sensitivity. Interference from several organic acids (e.g., tartaric) and divalent and trivalent cations.	[45] 2006
Spectrophotometry—oxalic acid replacement of DBS-arsenazo in Zr(IV) complex	9.27	0.815	Linear from 9 to 500 µM (r = 0.9995)	Within-run precision: 1.27% to 2.14% RSD Recovery: 99.2% to 99.6% Accuracy: results were comparable to method in [43]	Tomato sample was boiled in water for 30 min, cooled, and filtered.	An HCl solution containing zirconium(IV)-(DBS-arsenazo) was prepared. Oxalic acid replaces the DBS-arsenazo to produce a hyperchromic effect at 520 nm.	Simple sample preparation	High limit of detection. Interference from several organic acids (e.g., salicylic, tartaric and malic) and divalent and trivalent cations.	[46] 2008
Spectrophotometric—oxalate catalysis of crystal violet oxidation by dichromate	0.57	0.05	Linear from 0.2 to 5.5 µg/mL (r = 0.9964)	Within-run precision: 1.8% to 6.0% Recovery: 92% to 108% for tap water, and 96% to 115% for foods. Accuracy: results were comparable to a standard method (standard method not described)	Spinach or mushroom were pulverized in a mortar, boiled in water for 45 min, and filtered. Iron cations were removed by alkalinization to pH 10 and centrifugation. The solution was neutralized with HCl.	A solution of crystal violet, potassium dichromate, and sulfuric acid was combined with the oxalate-containing sample. The increase in absorbance intensity at 630 nm was recorded from 30 to 250 s.	Sensitive. Simple sample preparation.	Interference from divalent and trivalent cations (reduced by precipitation as metal hydroxides). Interferences form other organic anions not tested.	[47] 2009
Spectrophotometric—oxalate sequesters Cu(II) ion from Reactive Blue 4	0.62	0.055	2.07 Linear from 1.76 to 49.4 µM (R^2^ = 0.9983)	Within-run precision: 0.8% to 2.7% RSD Recovery: 97.9% to 101.2% Accuracy: results were comparable to reference method (reference method used not reported)	Urine was used directly. Mushroom and spinach samples were pulverized in a mortar, diluted with water, boiled, and filtered. NaOH was added to remove iron cations (centrifugation) prior to neutralization with HCl.	Reactive Blue (RB4) was complexed with Cu(II) in 10 mM HEPES buffer (pH 7). Oxalate-containing samples were added, and absorbance increase at 607 nm was monitored.	Simple sensor production from commercially available RB4. Simple sample preparation. <1 min response time.	Selectivity for oxalate was tested only against ascorbic acid and inorganic anions, not other multivalent organic anions (e.g., citrate and succinate)	[48] 2018
Spectrophotometric—oxalate inhibition of reaction of curcumin nanoparticles with Fe(III)—colorimetric	0.87	0.077	1.70 1.70 to 19.32 µM (r = 0.996)	Within-run precision: RSD = 4.27% (0.40 µg/mL oxalate) and 2.71% (1.05 µg/mL oxalate) Recovery: 95–105% Accuracy: not compared to other methods, but known mass of oxalate added yielded 95–105% recovery.	Food was cut and crushed to form a paste that was then boiled in water for 45 min. Filtrate was diluted with water to 100 mL. pH was adjusted to 10 with NaOH to eliminate interference from iron cations. Solutions were centrifuged, and supernatant decanted and neutralized with HCl. River water samples were filtered before adding HCl to remove carbonate and bicarbonate, then filtered again and neutralized with NaOH. Urine was diluted with water and used directly without further preparation.	Oxalate inhibited the reaction of curcumin nanoparticles (CURN) with Fe (III) in acidic media. Oxalate was measured by absorbance of CURN with Fe (III) at 427 nm. Absorbance intensity was linear with oxalate from 0.15 to 1.70 ug/mL. The method was used for detection of oxalate in water, food, and urine.	Sensitive and simple. Effective for water, food, and urine samples.	Interference from copper and carbonate/bicarbonate. Unknown if there is interference from other substances.	[49] 2018
Spectrophotometric—oxalate sequestration of Zr(IV) from zirconium	3.41	0.3	Linear from 1.0 to 6.0 µM (R = 0.997)	Within-run precision = 2.6% CV Recovery: 83% to 108%	Tea was prepared, filtered, and diluted in water prior to analysis.	Three solenoid micropumps (10–40 µL per pump) were coupled with a multi-channel CCD spectrophotometer. 1,8-dihydroxy-2-(4-sulfophenylazo)-naphthalene-3,6-disulphonic acid and oxizirconium chloride were mixed in HCl to create red colored zirconium-SPADNS. Oxalate was able to complex with Zr(IV), decreasing absorbance at 570 nm.	Simple sample preparation.	Interference from organic and inorganic anions not tested (other than fluoride). Narrow linear range. Instrumentation requires technical skill.	[50] 2020
Fluorescence—CdTe-based fluorescence quenching by oxalate reduction of Cu^2+^	0.08	0.007	0.100 0.100 to 1 × 10^4^ µM (R^2^ = 0.995)	Precision not reported, but linearity demonstrated from 100 nM to 10 mM. Recovery: 96–105% Accuracy: results were similar to LC-MS (r^2^ not reported)	Urine samples were centrifuged, supernatant was transferred and diluted 200 × with water. 40 µL of diluted sample was mixed with a solution containing 5 µL CuSO_4_ (100 µm) and 50 µL 10 mM MOPS buffer (100 mM NaNO_3_, 2.5 mM Mg(NO_3_)_2_, pH 7.4). Then, 1 µL CdTe quantum dot solution was added and incubated for 2.5 min. The solution was diluted with 200 µL water before fluorometry (365 nm ex., 570–720 nm em.)	Cadmium telluride (CdTe) fluorescence intensity was measured in urine samples. Oxalate-mediated reduction of Cu^2+^ to Cu^+^ increased quenching of CdTe fluorescence, as Cu^+^ was a more efficient quencher than Cu^2+^. The method was able to distinguish between urine with calcium oxalate stones or uric acid stones.	Simple protocol with low technical skill requirement. High sensitivity and upper limit of quantitation.	Not tested for interference with other organic anions found in urine (e.g., citrate)	[51] 2020
Fluorescence—carbon dots	0.65 (reported LOD, but data shows no response to oxalate below 11.37 µM)	0.057 (reported LOD, but data shows no response to oxalate below 1.0 µg/mL)	Linear from 11.4–227 µM (r = 0.9994)	Within-run precision: 0.14% to 0.34% RSD Recovery: 91.07–105.3%	Tomato or cherry samples were cut up and boiled in water, then filtered and diluted with water, and used directly for the assay.	Fluorescent carbon dots were produced from wild jujube and DL-tryptophan by heating in water for 9 h at 170 °C. Hg^2+^ caused static quenching of fluorescence (ex. 365 nm, em. 455 nm), which was recovered by sequestration of Hg^2+^ by oxalic acid.	Simple sample preparation and instrumentation.	Reported LOD not consistent with data shown. Chemically undefined wild jujube may lead to variable carbon dot fluorescence.	[52] 2022
Spectrophotometric—Fe(III)-sulfosalicylate-based colorimetric chemosensor	8.41	0.74	Linear from 0.80–160 µg/mL (r = 0.999)	Within-run precision ≤1.88% RSD Recovery = 98.99% to 104.53%	Oxalic acid-containing model wastewater was mixed with P25 TiO_2_ and subjected to simulated sunlight and ozone. Catalyst was removed, and assay was performed directly on solutions.	505-nm light absorbance by Fe(III)-sulfosalicylate was measured. Absorbance decreased proportionally with an increase in oxalate.	Interference from malonic acid, methylmalonic acid, and succinic acid was tested. No interference from various inorganic cations and anions.	Some reduction in fluorescence was caused by malonic acid and methylmalonic acid, albeit with less potency than oxalic acid.	[53] 2023

## Data Availability

No original data reported.
